# Artificial light impacts the mate success of female fireflies

**DOI:** 10.1098/rsos.220468

**Published:** 2022-08-10

**Authors:** Avalon C. S. Owens, Sara M. Lewis

**Affiliations:** Department of Biology, Tufts University, Medford, MA 02155-5801, USA

**Keywords:** light pollution, artificial light at night, Lampyridae, *Photinus*, firefly, mate success

## Abstract

Anthropogenic light pollution is a novel environmental disruption that affects the movement, foraging and mating behaviour of nocturnal animals. Most of these effects are sublethal, and their net impact on reproductive fitness and population persistence is often extrapolated from behavioural data. Without dedicated tracking of wild individuals, however, it is impossible to predict whether populations in light-polluted habitats will decline or, instead, move to shaded refuges. To disentangle these conflicting possibilities, we investigated how artificial light affects mating and movement in North American *Photinus*, a genus of bioluminescent fireflies known to experience courtship failure under artificial light. The degree to which artificial light reduced mate success depended on the intensity of the light treatment, its environmental context, and the temporal niche of the species in question. In the laboratory, direct exposure to artificial light completely prevented mating in semi-nocturnal *Photinus obscurellus*. In the field, artificial light had little impact on the movement or mate success of local *Photinus pyralis* and *Photinus marginellus* but strongly influenced mate location in *Photinus greeni*; all three species are relatively crepuscular. Our nuanced results suggest greater appreciation of behavioural diversity will help insect conservationists and dark sky advocates better target efforts to protect at-risk species.

## 1. Introduction

Wild organisms everywhere face an expanding array of anthropogenic threats that can disrupt their access to resources vital for survival and reproduction (e.g. habitat loss, water and air pollution, nutrient dilution) [1]. Few are both as pervasive and unprecedented as artificial light at night (ALAN) [2]. Recent estimates suggest that at least 50% of the habitable land surface of the Earth is illuminated by artificially bright night skies [2,3], which have become at least 49% brighter over the past 25 years following the development of increasingly inexpensive and efficient lighting technologies [4,5]. The intensification of artificial light is probably problematic to some degree for all organisms that evolved under predictable cycles of light and dark [6]. However, its effects should be especially strongly felt by nocturnal species that have evolved over millions of years to seek shelter, food and conspecifics in natural darkness.

Lethal effects of ALAN on these species have long been documented: moths and other insects swarm open flames [7,8], migrating seabirds collide with lighthouses [9,10], deer freeze in headlights [11,12], and sea turtle hatchlings crawl into oncoming traffic [13,14]. In recent decades, as ALAN has gained legitimacy as a conservation threat [15], researchers have further documented sublethal effects on the foraging [16,17], courtship [18,19] and mate success [20,21] of diverse taxa, with cascading consequences for ecological communities [22–26]. However, there remains a missing link between individual fitness and population persistence. Local extinction and mass emigration can be conflated, and, because ALAN often strongly affects movement, we are in many cases unable to predict whether affected populations will decline or simply go elsewhere [15,27].

Artificial light is particularly disruptive to fireflies (Coleoptera: Lampyridae), charismatic beetles that have attracted popular and scientific interest for millennia [28]. All of the 2200 species worldwide produce bioluminescent warning signals as larvae, which they use to inform predators of their unpalatability [29,30]. Adults of most species have since co-opted this bioluminescent ability for courtship signals: glows or patterns of flashes used to attract mates during the brief adult breeding season [31,32]. Roving flashing fireflies engage in courtship dialogues in which patrolling males emit discrete periodic advertisements from the air that prompt precisely timed responses from females on the ground [33]. Both male flash patterns and female response delays are species-specific to prevent hybridization [34,35]. Courtship dialogues are thought to be essential for mate success in nocturnal fireflies, as the males of most species are presumed not to use reflectance-based visual (colour) or chemical (pheromone) cues and thus have no other method of locating receptive females [36].

Recent research on flashing fireflies has found that patrolling males are less abundant [37,38] and/or less active [18,39,40] near artificial lights. Stationary female fireflies under artificial lights are even more strongly affected, rarely if ever answering nearby male advertisements [18,40]. Reduced courtship signalling is assumed to translate directly to decreases in mate success, which would justify ALAN as one of the top threats to firefly conservation [41]. However, several other possible scenarios exist. Males may still be capable of locating non-responsive females under artificial light by means of reflectance-based visual or even chemical cues, at which point the pair may or may not mate. Remarkably, few studies to date have directly explored the impact of ALAN on firefly mate location or mate success [20,40]. A growing body of research on the European glow-worm, *Lampyris noctiluca*, suggests that males are less likely to approach continuously glowing imitation females (green LEDs) placed beneath artificial lights [42,43], but the impact of ALAN on female attractiveness has yet to be investigated in any flashing firefly species.

If, for whatever reason, fireflies are unable to mate in the vicinity of artificial light, that does not necessarily imply that an artificially illuminated population will become locally extinct. Instead, adults might aggregate within dark refuges, just as advertising males limit their flash activity to forest edges in early twilight and, anecdotally, under full moonlight [44,45]. Notably, however, recent research suggests that some males may be attracted to rather than repelled by artificial lights [40]. Female movement is more relevant to predictions of population persistence [46], but considerably less well-understood. Estimates of the lifetime dispersal of adult females are rare, but can range from 10 m at most for *L. noctiluca* [47] to 100 m on average for *Luciola cruciata* (Ohba 1988, reported in [48]), and are probably contingent on ecological and morphological factors (e.g. habitat specificity and female flightlessness) [49]. Flightless *L. noctiluca* females have recently been shown to exhibit neither negative nor positive phototaxis, even when moving from illuminated to dark display sites would dramatically increase their chances of attracting males [50]. The phototactic tendencies of flashing female fireflies remain unknown.

In this paper, we seek to fill these various knowledge gaps by directly observing the impact of representative intensities of broad-spectrum artificial light on the reproduction of multiple species of North American *Photinus* firefly. First, we investigate whether ALAN affects the attractiveness of receptive *P. greeni* females, which we simulate using flashing green LEDs, to patrolling males. Next, we ask whether ALAN affects the probability that *P. obscurellus* pairs in close proximity will mate successfully by observing their courtship and mating behaviour in the laboratory and field. Finally, we document the mate success and movements of *P. pyralis* and *P. marginellus* females marked and released at varying distances from a permanent artificial light source in a patchily illuminated field. Together, these studies aim to provide insight into the long-term consequences of ALAN for at-risk species.

## 2. Methods

### 2.1. Female attractiveness

To investigate whether female fireflies positioned under artificial lights (5 lux) are less likely to attract patrolling males, we conducted binary choice trials on free-flying *P. greeni* males at sites in Lincoln (42.4259, −71.3164; 1090 µcd m^−2^ artificial night sky brightness; lightpollutionmap.info [3]) and Concord (42.4455, −71.3726; 836 µcd m^−2^), MA, between 23 June and 13 July 2020. In this region, *P. greeni* adults can frequently be observed at twilight (14.2 ± 4.0 min past sunset; mean ± s.d., *n* = 12 survey evenings) displaying along pathways in closed forests [51,52].

Observers answered periodic courtship advertisements from patrolling males with two identical response flashes simultaneously produced by a matched pair of programmed LED imitation females (electronic supplementary material, figure S1), only one of which was illuminated to 5 lux at ground level (simulating severe skyglow [53] or a dim or distant streetlight [54]) by a white LED flashlight (Alonefire) suspended from a tripod. When a dialoguing male flew within 40 cm of either imitation female, he was netted and his choice was recorded. At the end of each evening, captured males were marked with non-toxic fluorescent powder and released. None were recaptured in subsequent trials. For additional methods, see electronic supplementary material, text S1.

The preference of patrolling *P. greeni* males for approaching either dark control or artificially illuminated imitation females was compared with an unbiased random choice model (where *P*(event) = 0.5) using an exact binomial test. This and all following analyses were conducted in RStudio (v. 1.4.1103).

### 2.2. Mate success in the laboratory

To investigate how artificial light affects mate success in fireflies, we observed 56 pairs of male and female *P. obscurellus* fireflies in darkness (less than 0.01 lux, hereafter reported as 0 lux) or under dim (3 lux) or bright (30 lux) illumination in the laboratory. Individuals were collected periodically between 29 May and 27 June 2020 from sites in Lincoln, MA, USA (42.4259, −71.3164, 1090 µcd m^−2^; 42.4258, −71.3066, 1170 µcd m^−2^), where they were found in low wet fields (see [18] for more details on collection methods; [51,52]). Two pairs were tested under the same treatment every evening between 2 and 29 June 2020 with the treatments cycled haphazardly throughout the month to control for seasonal variation in mate choice [55].

Trials took place in an enclosed porch illuminated during the day by a single screened window open to outside air (electronic supplementary material, figure S2a). Two matching enclosures (60 cm^3^) were placed side-by-side directly beneath this window. A removable semi-cylindrical arena (22 × 28 cm) made of transparent acrylic was superimposed upon the central wall of each enclosure, and the open top of each enclosure covered with a layer of white diffusion fabric. Enclosures were thus exposed both to natural twilight from the adjacent window (with sunset falling between 20.17 and 20.27) and, on experimental evenings, to artificial light from an overhead white LED bulb (Shabulb) filtered to either 3 or 30 lux. At 19.30, one male and one female were gently introduced into each arena through separate entrances. On experimental evenings, the overhead artificial light switched on automatically at 20.00. Infrared cameras (Raspberry Pi Zero W) mounted on the walls opposite each arena took one photo of the pair per minute from 19.30 to 7.00 the next day. Photos were manually scored to obtain the time at which pairs initiated mating stages one (mounting) and two (spermatophore transfer) [56,57,58], as well as the duration of each mating stage (electronic supplementary material, figure S2b). Pairs were categorized as successfully mated only if they reached mating stage two (following [58]). For additional methods, see electronic supplementary material, text S1.

The impact of artificial light on mate success was assessed using a generalized linear model (GLM) with a binomial distribution that initially included fixed effects of trial date, collection site, enclosure and arena as well as treatment (0 lux, 3 lux or 30 lux, measured at the bottom of both enclosures in complete darkness). The impact of artificial light on the time at which pairs initiated mating (in minutes past sunset) and on the duration of mating stage two were assessed using separate linear models with the same fixed effects. Variation in mating times among treatments was checked for homogeneity of variance using Levene's test. The full models were competed with their simpler nested counterparts using Akaike's information criterion (AIC), and fixed effects that did not significantly improve model fit were dropped. The significance of the remaining fixed effects was assessed via likelihood ratio (LR) tests [59]. Mate success under either light treatment (3 lux or 30 lux) was compared with the rate in the dark control treatment (0 lux) using two Bonferroni-corrected exact binomial tests.

### 2.3. Mate success in the field

To investigate whether artificial light inhibits firefly mate success in more visually complex natural habitats, we observed 34 pairs of *P. obscurellus* fireflies in the field, some illuminated and some left dark, on 14 evenings between 1 June and 20 June 2020. We searched sites in Lincoln, MA, USA (see above) for females actively dialoguing with one or more nearby males. A dedicated observer approached each candidate female, suspended a white LED flashlight (Smith–Wesson, filtered to 5 lux at 55 cm distance, ground level) from a tripod directly above her display perch, and crouched nearby. When a male landed or walked within 5 cm of the focal female, the observer either switched the flashlight on manually to initiate an experimental trial, or imitated the same motion to initiate a control trial. During each trial, the observer took audio notes on the behaviours of the focal female and all nearby males until mating occurred, no males were seen within approximately 1 m of the female, or 15 min had passed (following [57]). For additional methods, see electronic supplementary material, text S1.

The impact of artificial light on mate success was assessed using a binomial GLM including effects of treatment (0 lux or 5 lux), trial date, trial start time (in minutes past sunset) and observer. The impact on male courtship flash activity (flash patterns per minute) was assessed using a linear model with the same effects. The impact on female receptivity was investigated in two ways. The rate at which females answered advertising males was assessed using a Poisson GLM in which counts of female response flashes were set against the same effects and counts of male advertisements included as an offset. The proportions of single, double or triple response flashes produced by females in the two treatments were compared using a *χ*^2^ test of independence. Finally, a binomial GLM that included effects of female response rate and proportion of single response flashes, but not treatment, was used to assess which was more strongly linked to mate success. Models were evaluated as described above.

### 2.4. Female movement

To investigate whether female fireflies in light-polluted habitats move away from artificial lights and toward dark refuges over time, and to understand the consequences of such movement for reproduction, we collected and then relocated 150 *P. pyralis* and 66 *P. marginellus* females in Tionesta, PA, USA to dark (0 lux), dim (less than 2 lux) and bright (greater than 20 lux) release sites, then tracked their subsequent movements and mate success between 6 July and 30 July 2019. Teams of two to five trained researchers caught females of both species in a mown field behind the Tionesta Lake Visitor Center, maintained by the US Army Corp of Engineers since the mid-1980s. In late 2015, three white LED security lights were installed on the southwest face of the visitor centre (B Parkhurst 2022, personal communication). These lights illuminate nearly half of the back field (an estimated 75 m swathe). They switch on automatically at sunset and remain on throughout the *P. pyralis* and *P. marginellus* courtship activity period. Both of these species are active at sunset and produce exclusively single flashes, but are easily distinguished by body size and lantern morphology [44,51]. The females of both species are winged but rarely fly [60].

The location of each collected female was marked with a flag. Near the end of each evening, collected females were mass-marked with a combination of non-toxic fluorescent powder and gel pen to signify their capture date. They were then released at one of 14 GPS-tagged locations (figure 3*a*): four bright release sites on the area of lawn nearest to the security lights (greater than 20 lux; 2.2 ± 0.2 m from the closest source), four dim release sites (less than 2 lux; 17.6 ± 3.7 m) and six dark release sites (0 lux; 83.6 ± 19.4 m). A single dedicated observer systematically surveyed every release site throughout the evening, searching for marked females using a UV flashlight. Resighted females were flagged and their current mating status (paired or unpaired) recorded. As spermatophore transfer can take 20 min or more in *Photinus* species, and surveys spanned the entire courtship activity period, this method probably accurately assessed the nightly mate success of all resighted females. The following afternoon the distance and angle of each flag with respect to its nearest GPS-tagged landmark was measured, and the flags removed. For additional methods, see electronic supplementary material, text S1.

The coordinates of each capture and resight location, as well as its distance from the nearest light source, were calculated from these values and mapped in R (WGS84) [61,62]. Artificial illumination at each unique location was estimated from lux measurements taken at release sites via inverse square distance interpolation in Google Earth Engine [63]. The movements of marked females were modelled following methods from Iles *et al.* [64]. Briefly, gamma, exponential, Weibull and lognormal probability distributions were fit to the set of observed dispersal distances using maximum-likelihood estimation [65] and the support for each compared via AIC. Based on the outcome, a lognormal GLM was used to model effects of species, evenings post-release and their interaction on the displacement distance of females from release sites. The probability of resighting females at all at the dim and bright release sites was compared with that at dark release sites via two Bonferroni-corrected exact binomial tests.

To investigate the phototactic tendencies of resighted females, their change over time in average distance to the nearest light source and interpolated intensity of artificial illumination were assessed using separate linear mixed models. Both models contained fixed effects of species, release site brightness (0 lux, less than 2 lux or greater than 20 lux), number of evenings post-release, and all possible pairwise interactions, as well as a random effect of release group. Finally, a binomial generalized linear mixed model (GLMM) [66] with fixed effects of species, release site brightness, distance to the nearest light source, and interpolated artificial illumination, as well as a random effect of release group, was used to assess the degree to which these variables influenced the probability of mating successfully. Models were evaluated as described above.

## 3. Results

### 3.1. Female attractiveness

Across all 12 evenings of *P. greeni* binary choice trials, 28 out of 29 lured males (96.6%) preferentially approached the dark control imitation female (0 lux) rather than the imitation female that was illuminated by artificial light (5 lux; 95% CI: 82.1–99.9%; exact binomial test, *p* < 0.0001; electronic supplementary material, figure S3a).

### 3.2. Mate success in the laboratory

**Figure 1.**
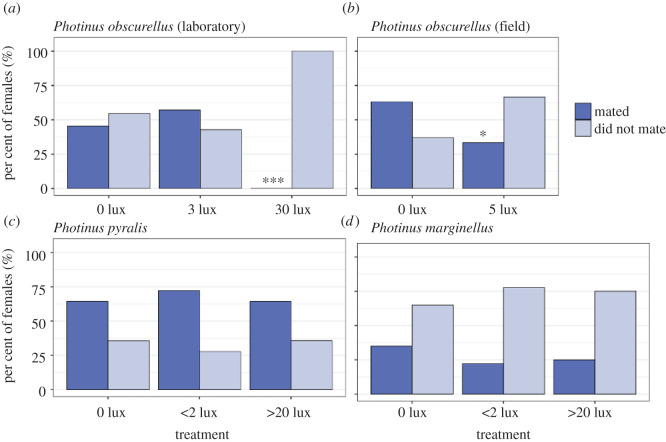
Impact of artificial light at night on the mate success of three *Photinus* species in the laboratory (*a*) and in the field (*b,c*). Asterisks indicate statistically significant differences compared with the dark control (exact binomial test; * = *p* < 0.05; *** = *p* < 0.0001). (*a*) Mating outcomes among 56 *P. obscurellus* pairs confined together for 11.5 h under natural darkness from a nearby open window (0 lux), dim artificial light (3 lux) or bright artificial light (30 lux). (*b*) Mating outcomes among 34 *P. obscurellus* pairs that were actively courting at close distance when an unlit (0 lux) or lit (5 lux) flashlight was placed directly above the female. (*c*) Mating outcomes of 119 *P. pyralis* females resighted one or more days after their release at dark (0 lux), dim (less than 2 lux) or bright (greater than 20 lux) release sites. (*d*) Mating outcomes of 59 *P. marginellus* females resighted one or more days after release at the same release sites.

Artificial light significantly impacted the mate success of *P. obscurellus* pairs in the laboratory (figure 1*a*; binomial GLM; treatment: LR *χ*^2^ = 35.10, d.f. = 2, *p* < 0.0001). In natural twilight, 10 out of 22 pairs (45.5%) mated successfully. Under 3 lux of artificial light, 8 out of 14 pairs (57%) mated successfully, a non-significant difference (45.5% versus 57%: *p* = 0.4288, exact binomial test). Under 30 lux of artificial light, however, not one of 20 pairs mated successfully (45.5% versus 0%: *p* < 0.0001). Mate success declined significantly over the course of the *P. obscurellus* mating season as well (date: LR *χ*^2^ = 17.13, d.f. = 1, *p* < 0.0001), regardless of treatment.

There was a marginally significant trend towards greater variation in the amount of time it took for successfully mating pairs to reach mating stage two (spermatophore transfer) under 3 lux of artificial light (electronic supplementary material, figure S3b; Levene's test; *F* = 3.64, d.f. = 1, *p* = 0.0745). However, artificial light did not impact the duration of mating stage two (linear model; treatment: *F* = 0.35, d.f. = 1, *p* = 0.5622) nor how long males spent dorsally mounting females beforehand (*F* = 0.92, d.f. = 1, *p* = 0.3514). Across treatments, pairs that mated successfully spent 1.2 ± 1.2 min in mating stage one and 20.7 ± 4.9 min in mating stage two (mean ± s.d.).

### 3.3. Mate success in the field

**Figure 2.**
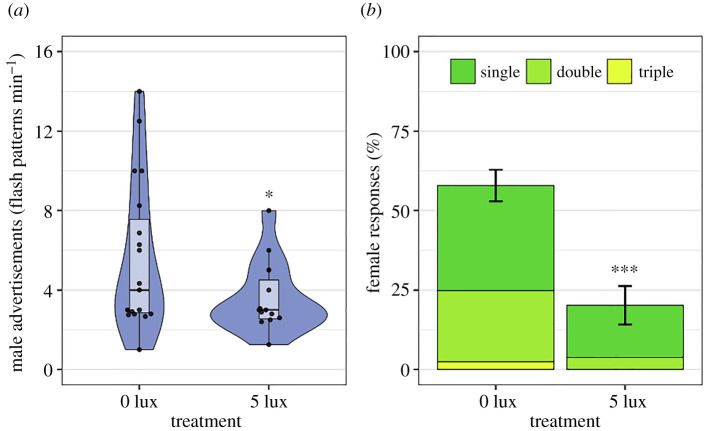
Impact of artificial light at night on the courtship dialogues of *P. obscurellus* fireflies in the field. (*a*) Male courtship flash activity near focal females. Asterisk denotes a statistically significant difference from the dark control (0 lux; binomial GLM; * = *p* < 0.05). (*b*) Female response rates, or the percentage of nearby male advertisements to which females replied, and the distribution of response flash patterns (single, double or triple). Asterisks denote a statistically significant difference in female response rates compared with the dark control (offset Poisson GLM; *** = *p* < 0.0001).

In the field, artificial light also significantly impacted the mate success of *P. obscurellus* pairs (figure 1*b*; binomial GLM; LR *χ*^2^ = 4.95, d.f. = 1, *p* = 0.0261). When actively dialoguing pairs were exposed to 5 lux of artificial illumination, overall male courtship flash activity declined significantly (figure 2*a*; LR *χ*^2^ = 5.02, d.f. = 1, *p* = 0.0250). Female response rates declined significantly as well (figure 2*b*; offset Poisson GLM; LR *χ*^2^ = 75.65, d.f. = 1, *p* < 0.0001), as did the proportion of double and triple (versus single) response flashes females produced (from 38.8% to 18.8% and from 4.1% to 0%, respectively; *χ*^2^ = 20.00, d.f. = 2, *p* = 0.0005). Female response rate was a stronger predictor of mate success than female response flash pattern (response rate: LR *χ*^2^ = 4.27, d.f. = 1, *p* = 0.0388; proportion single flashes: LR *χ*^2^ = 0.49, d.f. = 1, *p* = 0.4825; Pearson's *r* = −0.62; electronic supplementary material, figure S4).

Male activity, female receptivity and mate success were all significantly lower both earlier in the season (male activity: LR *χ*^2^ = 4.28, d.f. = 1, *p* = 0.0387; female response rate: LR *χ*^2^ = 3.55, d.f. = 1, *p* = 0.0595; mate success: LR *χ*^2^ = 6.58, d.f. = 1, *p* = 0.0103) and later in the evening (male activity: LR *χ*^2^ = 4.80, d.f. = 1, *p* = 0.0285; female response rate: LR *χ*^2^ = 4.19, d.f. = 1, *p* = 0.0407; mate success: LR *χ*^2^ = 9.13, d.f. = 1, *p* = 0.0025) across treatments.

### 3.4. Female movement

**Figure 3.**
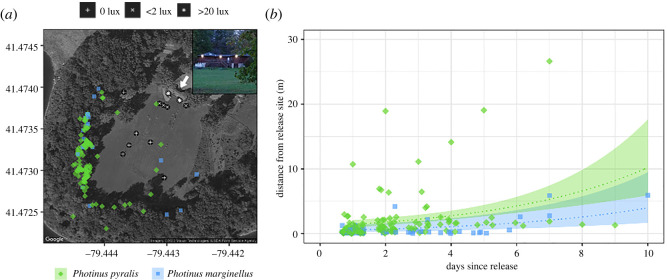
Initial distribution and movement of female fireflies within an artificially illuminated landscape. (*a*) Satellite map of the field behind Tionesta Lake Visitor Center (white arrow), with latitude and longitude displayed on the *y*- and *x*-axes, respectively. Semi-transparent points mark locations where 150 *P. pyralis* (green diamond) and 66 *P. marginellus* (blue square) females were captured over July 2019. The 14 locations where females were subsequently released are marked in black, with white symbols corresponding to their artificial light level: dark (0 lux), dim (less than 2 lux) or bright (greater than 20 lux). Three security lights mounted on the southeast face of the visitor centre (inset), less than 1 m from bright release sites, were the most prominent source of artificial illumination. Photograph courtesy Bruce Parkhurst. (*b*) Distance of resighted females from their release site over time. Females that died prior to resighting are not included as the number of days over which they moved is unknown. Because females were mass-marked and not recaptured, some individuals were probably resighted multiple times.

More than a third of the *P. pyralis* (38%) and *P. marginellus* (42%) females captured, marked and released were resighted the next evening, including most of those assigned to dark release sites (43 of 84, or 51.2%). Resighting rates were significantly lower at dimly lit release sites (19 of 59, 32.2%; *p* = 0.0078, exact binomial test) and lower still at brightly lit release sites (19 of 73, 26.0%; *p* < 0.0001). Resighting rates declined over subsequent evenings while the average distance of females from their release sites increased (figure 3*b*; lognormal GLM; LR *χ*^2^ = 18.77, d.f. = 1, *p* < 0.0001). The longest distances moved were 26.6 m over seven days for *P. pyralis* and 19.1 m over at most 21 days for *P. marginellus*; the latter female was dead when resighted. *Photinus pyralis* moved farther on average than did *P. marginellus* (LR *χ*^2^ = 9.65, d.f. = 1, *p* = 0.0019), but there was no interaction between species and the number of evenings post-release (LR *χ*^2^ = 1.17, d.f. = 1, *p* = 0.2791). The longest time elapsed between release and resighting was 10 days for a *P. marginellus* female found alive 5.9 m from her release site.

**Figure 4.**
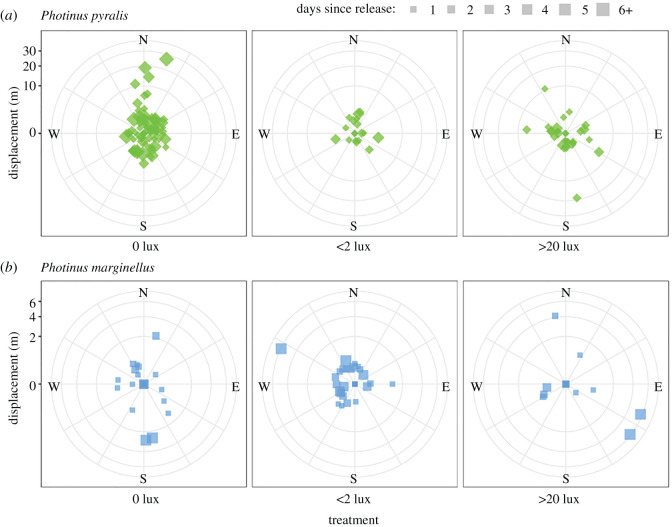
Impact of artificial light at night on the movement of *P. pyralis* and *P. marginellus* females in the field. (*a*) Displacement distance (in metres) and angle of *P. pyralis* females from their release sites (origin) over time. The size of each point corresponds to the number of days after release that each was measured. The *y*-axis has been square-root-transformed to better visualize shorter displacements. Security lights faced southwest, as shown in the previous figure. (*b*) Displacement of *P. marginellus* females from their release sites over time. Note the shorter *y*-axis scale, also square-root-transformed.

Across species, females released at bright sites were resighted 1.24 ± 3.28 m (mean ± s.d.) farther away from the nearest light source in the evenings post-release (figure 4; linear mixed model; LR *χ*^2^ = 11.88, d.f. = 2, *p* = 0.0026). Those released at dim and dark sites moved randomly with respect to the security lights (multiple comparisons, Tukey adjustment: greater than 20 lux versus 0 lux: *p* = 0.0128; greater than 20 lux versus less than 2 lux: *p* = 0.0221; less than 2 lux versus 0 lux: *p* = 0.9986). Females released at bright sites moved to locations that were an estimated 1.48 ± 4.30 lux dimmer on average (linear mixed model; LR *χ*^2^ = 6.88, d.f. = 2, *p* = 0.0320), while those released at dim and dark sites did not move to significantly brighter or darker locations (multiple comparisons, Tukey adjustment: greater than 20 lux versus 0 lux: *p* = 0.0374; greater than 20 lux versus less than 2 lux: *p* = 0.0610; less than 2 lux versus 0 lux: *p* = 0.9999). There were no discernable temporal trends in either model (*p* ≥ 0.1849), nor were there any effects of species (*p* ≥ 0.7154) or interactions between any combination of species, release site and evenings post-release (*p* ≥ 0.0895).

*Photinus pyralis* females were significantly more likely than *P. marginellus* females to attract mates (figure 1*c,d*; 65.5% versus 27.1%; binomial GLMM; LR *χ*^2^ = 16.24, d.f. = 1, *p* < 0.0001). However, mate success was not measurably impacted by any of the above measures of artificial light exposure—release site brightness, distance to the nearest light source or interpolated intensity of artificial illumination—in either species (LR *χ*^2^ ≤ 1.45 and *p* ≥ 0.2290 in all cases).

## 4. Discussion

ALAN is known to have sublethal impacts on the fitness-related behaviours of diverse taxa, but few studies to date have looked at its consequences for reproduction, and still fewer have investigated whether said consequences can be avoided by directed movement into dark refuges. The research reported here was conducted on fireflies in the genus *Photinus*, which are known to alter their courtship behaviour in response to ALAN, to address this gap. Using artificial illumination similar in intensity to that of a dim streetlight, we found that ALAN decreased the attractiveness of imitation females to crepuscular *P. greeni* males. The same intensity of ALAN suppressed the mating activity of semi-nocturnal *P. obscurellus* pairs in the field, while a brighter intensity completely prevented mating in the laboratory. However, permanent artificial light sources had no apparent impact on mate success among the females of two other crepuscular species, *P. pyralis* and *P. marginellus*, although these females tended to move away from the most brightly illuminated areas of habitat. Our disparate results emphasize the subtle differences that distinguish taxa broadly categorized as nocturnal and suggest that, in particular, species-specific temporal niches and visual environments influence the vulnerability of fireflies to light pollution.

Our study is the first to extend the considerable body of work on how ALAN affects mate location in the continuously glowing European glow-worm, *L. noctiluca* [42,67,68], to a discretely flashing North American firefly species. In field trials, we found that 5 lux of artificial illumination deterred patrolling *P. greeni* males from approaching flashing imitation females. Similarly, Elgert *et al*. [50] placed pairs of glowing imitation females side-by-side and found that *L. noctiluca* males approached the imitation female under 15–20 lux in only one of 19 trials. In both studies, it is unclear whether males are entirely unable to see illuminated females or only perceive them as dimmer, and thus less attractive [69], than their unilluminated counterparts; in the latter case, if all nearby females are artificially illuminated, less choosy males may still be able to find mates. In a study without unilluminated competitors, however, Elgert *et al*. [43] found that *L. noctiluca* males are still significantly less likely to approach even the brightest imitation females under 7 lux of artificial light, suggesting that ALAN impacts the visibility of bioluminescent courtship signals and thereby mate location in fireflies.

Previous research indicates that larval fireflies limit their foraging activity under bright artificial illumination [16,46] but this study is the first to show that adult fireflies cease mating activity (in addition to courtship activity) as well, even when males and females are in close proximity. In the laboratory, 3 lux of artificial illumination had no discernable impact on mate success of *P. obscurellus* pairs but 30 lux of artificial illumination prevented mating completely. This failure was unlikely to be the result of impaired mate location as pairs were confined together in an otherwise empty arena and at several points our cameras recorded males crawling directly past or even over females without initiating mating stage one. Perhaps counterintuitively, this fitness impact may have little to do with the visibility of bioluminescent signals: ALAN also suppresses foraging and mating activity in moths [70], thought to result from its masking of the environmental light cues that regulate circadian patterns of activity across nocturnal taxa [71,72].

In field trials, 5 lux of artificial illumination reduced the mate success of *P. obscurellus* pairs, most likely by interfering with the ability of males to locate females. Most males continued signalling and searching (crawling and hopping, *sensu* [73]) following the introduction of ALAN, but were unable to find non-responsive females among the complex vegetation; two of the five males that mated successfully did so shortly after the trial began, while they were still close to the focal females. Extending the results of Firebaugh and Haynes [40,74] on *P. pyralis*, we found ALAN suppressed female response rates more than it did male flash activity (35% of dark control values for females versus 64% for males). This finding also corroborates our previous laboratory study showing that ALAN has sex-specific impacts on the courtship flash activity of *P. obscurellus* pairs physically (but not visually) isolated in an empty arena (10% for females versus 50% for males under 24–240 lux of artificial illumination) [18].

The divergent effects of 3–5 lux of artificial illumination on *P. obscurellus* mate success when assessed in the laboratory versus the field in this study (no effect versus a drop to 53% of dark control rates) may reflect that ALAN was introduced gradually (with twilight) in the former but suddenly in the latter: sudden exposure to bright light saturates the dark-adapted compound eyes of nocturnal insects (reviewed in [75]). Because female fireflies scan the air above for advertisements from flying males, they may be more likely to be blinded by downwelling sources. Alternatively, the divergent effects could be due to the slightly higher intensity of ALAN used in field trials. Three and five lux at ground level are perceptually indistinguishable, at least to humans [76], but illuminance is inversely related to distance from a light source. Pairs in the laboratory were confined to a minimum distance of at least 85 cm from the light source while females in the field were sometimes perched almost directly beneath (less than 10 cm away from) the light source. Females that mated successfully under ALAN often sought darker microhabitats by crawling towards the ground or beneath leaves as they continued dialoguing with males (as in [36]).

Our study of the long-term movement of *P. pyralis* and *P. marginellus* females released near permanent artificial lights (greater than 20 lux), among the first of its kind, also showed that females tended to move towards darker areas. This finding contradicts records of positive phototaxis among *P. pyralis* males and *Photuris* females [40] and of a lack of phototaxis in *P. pyralis* males [74] and *L. noctiluca* females [50]. However, we saw no impact of ALAN on female movement outside of bright release sites, suggesting that these species are at most mildly negatively phototactic. It is also possible that females disproportionately avoided approaching the security lights mounted on the visitor centre solely because these lights overlooked paved pathways that lacked suitable display sites. However, because resighting rates at bright release sites were approximately 50% of those at dark release sites, it is just as likely that missing females displayed strong negative phototaxis in the form of long-distance dispersal flights or hiding: Elgert *et al*. [50] found that 27% of *L. noctiluca* females retreat into dark refuges when exposed to ALAN, just as 8.3% of *Photuris* larvae burrow underground [46].

Paradoxically, the movements we were able to observe seemed to be of little consequence to reproductive fitness: the mate success of *P. pyralis* and *P. marginellus* females was similar across the full range of artificial light levels. These results contradict those of the experiments on *P. obscurellus* reported above as well as other studies [20,74]. Van den Broeck *et al*. [20] observed *L. noctiluca* females displaying near streetlights (less than or equal to 8.5 lux) and found that they failed to mate for a median of six and as many as 24 evenings of their brief mating season. Firebaugh & Haynes [74] tethered *P. pyralis* females beneath a bright floodlight (167 lux) and found that they rarely flashed and never mated. In a subsequent study, however, the same authors [40] found no significant difference in the mate success of *P. pyralis* females enclosed with males in artificially illuminated mesh tents (approx. 40 lux) [40]. In the laboratory, Thancharoen [77] also found that artificial light (0.05–0.3 lux) had no effect on the mate success of *Sclerotia aquatilis* pairs, although it may have prolonged their courtship.

Another crucial variable that differs across studies, in addition to artificial light intensity and complexity of the visual environment, discussed above, is the temporal niche of each firefly species. *Lampyris noctiluca* is fully nocturnal, while *P. obscurellus* is semi-nocturnal, and *P. greeni*, *P. pyralis* and *P. marginellus* are all fully crepuscular [44]. Crepuscular fireflies are adapted to engage in courtship and mating under ambient light and may therefore be more resilient to the introduction of ALAN into their habitat [78], perhaps even benefitting from an artificially extended twilight. Such pattern is already emerging from studies measuring the impact of ALAN on male courtship flash activity in different species (reviewed in [38]). However, whether this pattern is also reflected in its impacts on mate success remains to be conclusively demonstrated. Future research is necessary to improve our understanding of the movement and mate success of fully nocturnal fireflies, especially those species currently listed as threatened or data deficient [79].

This study was informed by growing awareness among conservationists that an understanding of movement is needed to guide conservation planning [27]. Movements of females are of particular import when estimating vital rates, especially among roving flashing firefly species, whose relatively mobile males are often present in excess. At our study site behind the Tionesta Lake Visitor Center, *P. pyralis* males could be seen displaying across the entire field (B Parkhurst 2022, personal communication), while females appeared mostly confined to the forest edge; *P. pyralis* and *P. marginellus* females released hundreds of metres from their collection site were still able to attract nearby males. While these species appeared relatively unaffected by ALAN, the decreased mate success exhibited by *P. obscurellus* pairs in the laboratory and the field is likely to culminate in population declines if affected females are unable to move into dark refuges.

Of course, if the availability of dark refuges continues to shrink [5], the question of whether female fireflies are capable of such movement will become irrelevant. Conservation management and educational outreach can help to combat the intensification of ALAN, especially within protected areas and biodiversity hotspots [80,81], and by doing so minimize its lethal and sublethal impacts on bioluminescent fireflies and other taxa in need of natural darkness.

## Data Availability

All data and code are included as .csv files in a zipped folder uploaded as electronic supplementary material [82].
